# Spontaneous rupture of primary splenic angiosarcoma: a case report and literature review

**DOI:** 10.1186/1477-7819-11-53

**Published:** 2013-03-04

**Authors:** Yun-Fei Duan, Yong Jiang, Chuan-Xing Wu, Feng Zhu

**Affiliations:** 1 Department of Hepatobiliary Surgery, The Third Affiliated Hospital of Soochow University, 185 Juqian Street, 213000, Changzhou, Jiangsu, China

**Keywords:** Spleen, Neoplasm metastasis, Sarcoma

## Abstract

**Background:**

Primary angiosarcoma of the spleen is a rare mesenchymal malignant tumor of vascular origin often with a poor prognosis, due to its high metastatic potential. This disease often presents with atraumatic rupture and lethal hemorrhage.

**Case presentation:**

We report a case of a 65-year-old man who presented with abdominal pain, anemia, thrombocytopenia, and palpable abdominal mass with unstable blood pressure. Laparotomy revealed a huge actively bleeding spleen, thus splenectomy was performed. Some liver metastasis foci were also found during the procedure. Histopathology diagnosis of the removed spleen was primary splenic angiosarcoma. The patient was discharged on the 10th day post operation with no complication.

**Conclusions:**

Splenic angiosarcoma should be considered one of the differential diagnoses in patients with spleen parenchymal lesions. Definitive diagnosis requires laparotomy followed by splenectomy. In the majority of the patients with spleen angiosarcoma, metastatic diseases have already occurred at the time of laparotomy, so splenectomy is an approach more for diagnostic purpose rather than curative purpose.

## Background

Primary angiosarcoma of the spleen is an extremely rare malignancy, the pathogenesis of which is unknown, with high metastatic potential and a very poor prognosis. This aggressive disease usually presents in adults in their sixth to seventh decade. The reported median survival rates range from 4.4 to 14 months [1,2]. These mesenchymal tumors can easily be neglected and splenic rupture is the most frequently manifestation at the time of diagnosis. We report a case of spontaneous splenic rupture due to angiosarcoma in a 65-year-old man.

## Case presentation

**Figure 1:**
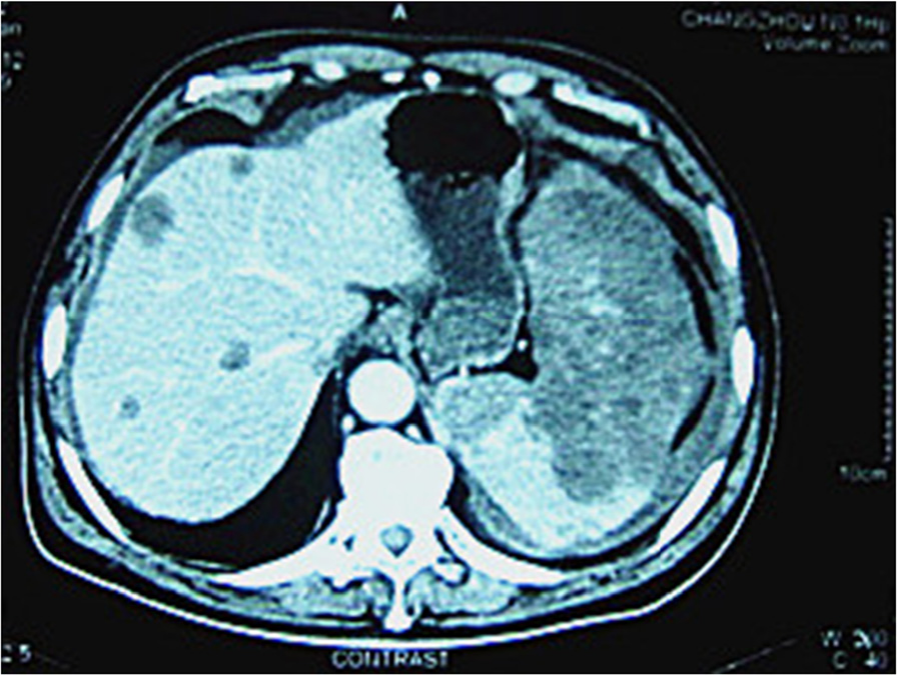
**Enlarged spleen completely replaced by low-density tumor tissue and multiple hypervascular metastases in liver.** Contrast-enhanced image in the portal venous phase computed tomography scan showing the enlarged spleen completely replaced by tumor tissue with low density and multiple hypervascular metastases in the liver with sizes varying from 5 mm to 2 cm. Some free fluid around the liver and spleen was also found, indicating intraperitoneal hemorrhage.

A 65-year-old man was admitted with diffuse abdominal pain and distention. The pain was constant and dull, and started 2 hours prior to admission with no obvious inducible factor. The patient’s past medical history was unremarkable except mild weight loss during the recent 5 months. Physical examination revealed a palpable abdominal mass in the left upper abdominal quadrant with tenderness, and the patient’s hemodynamic condition was unstable with systolic arterial blood pressure only 85 mmHg and heart rate 110 beats/minute. After initial fast fluid resuscitation (1,000 ml hetastarch 130/0.4 NaCl), the hemodynamic status of the patient became stable. Peripheral blood count revealed anemia (hemoglobin 10.0 g/dl) and thrombocytopenia (platelets 90 × 10^9^/l), but it did not reach disseminated intravascular coagulopathy. Results of other laboratory examinations had not been reported to us before the operation due to the emergency. Abdominal ultrasonography showed an enlarged spleen filled with irregular nodules, multiple hepatic solid lesions and moderate accumulation of peritoneal fluid. Abdominal paracentesis was performed with blood fluid aspirated from the peritoneal cavity. These findings were further confirmed by an abdominal computed tomography scan (Figure 1), demonstrating a heterogeneous, low-density signal within the splenic parenchyma, with variable degrees of contrast enhancement as well as intra-abdominal hemorrhage originating most possibly from the spleen. Meanwhile, there were multiple low-density lesions with enhanced rim in the liver.

Laparotomy revealed a huge spleen actively bleeding and an abnormal liver with several metastatic foci. We did observe a fissure of the capsule of the spleen with a size of 6 cm (length) × 2.5 cm (depth) in the lower pole of spleen. Two liters of blood had been collected from the peritoneal cavity. Splenectomy was performed. During the procedure, the patient received 4 units of concentrated red blood cells and 2 units of fresh frozen plasma.

**Figure 2:**
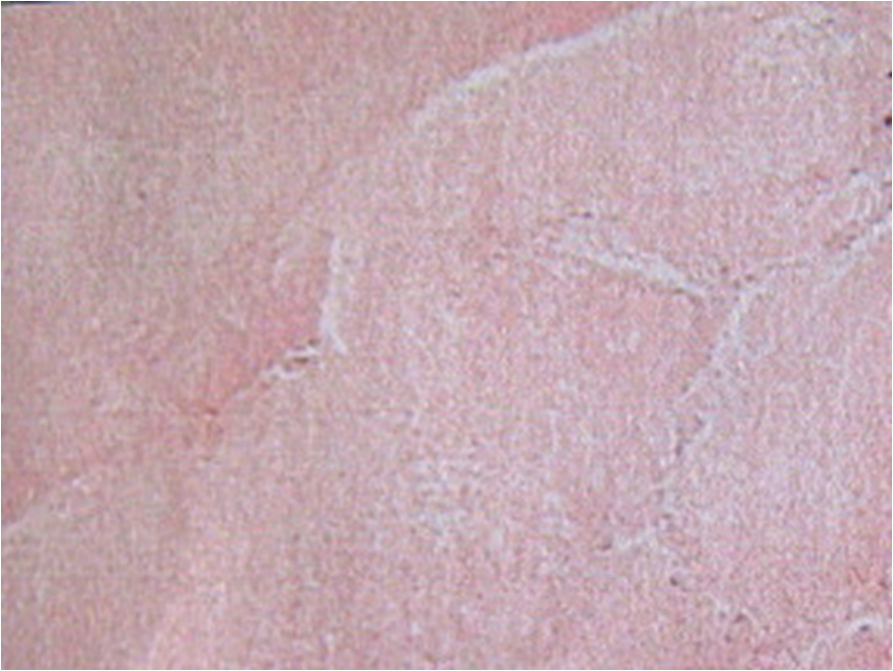
**Histopathologic findings of the spleen angiosarcoma.** Spindle tumor cells have replaced the normal red and white pulp in the spleen, whereas ecstatic vascular spaces lined with hypertrophied endothelial cells are apparent.

**Figure 3:**
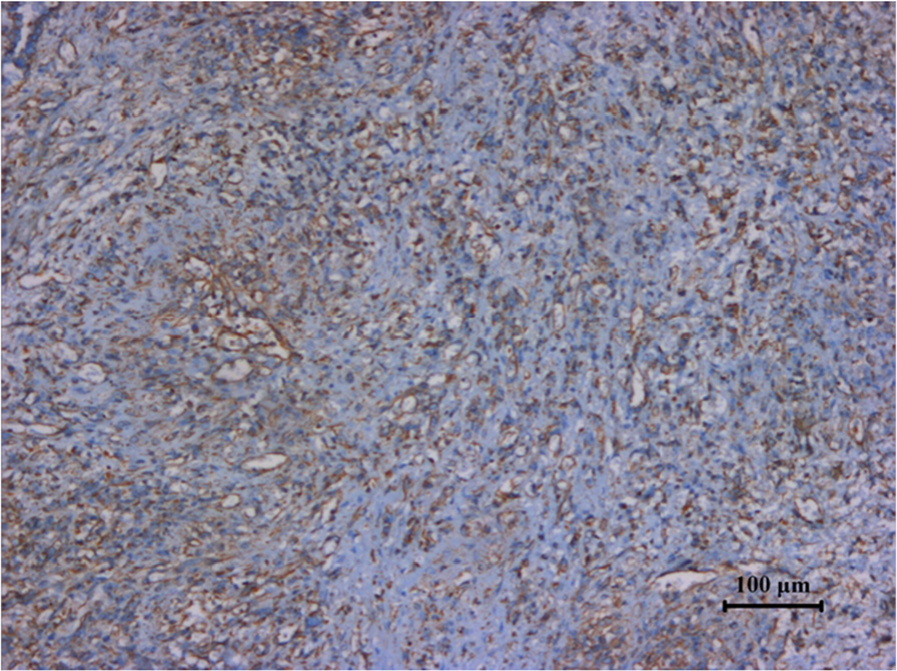
Specimen stained positive for vimentin.

**Figure 4:**
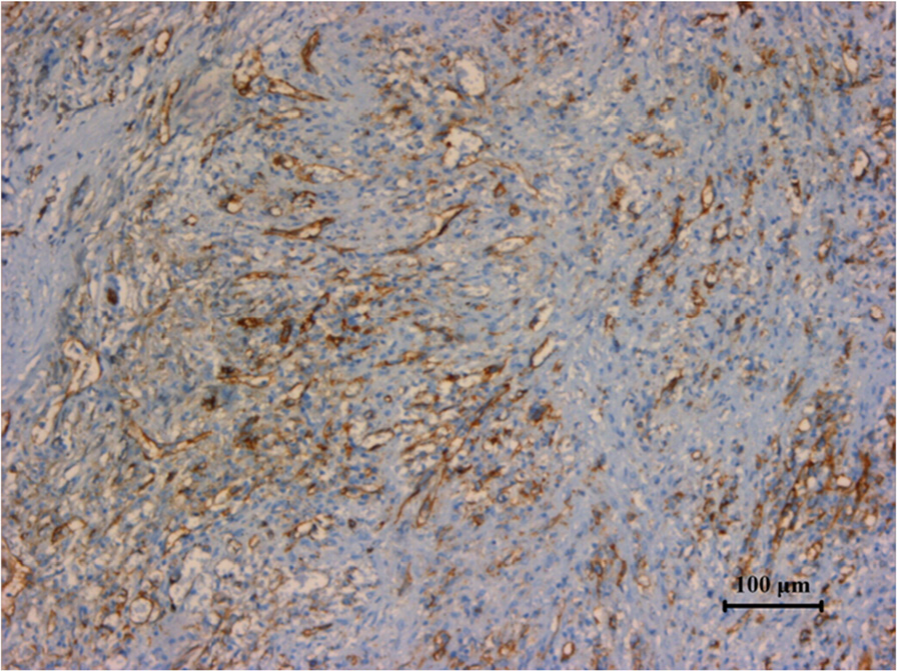
Specimen stained positive for CD31.

**Figure 5:**
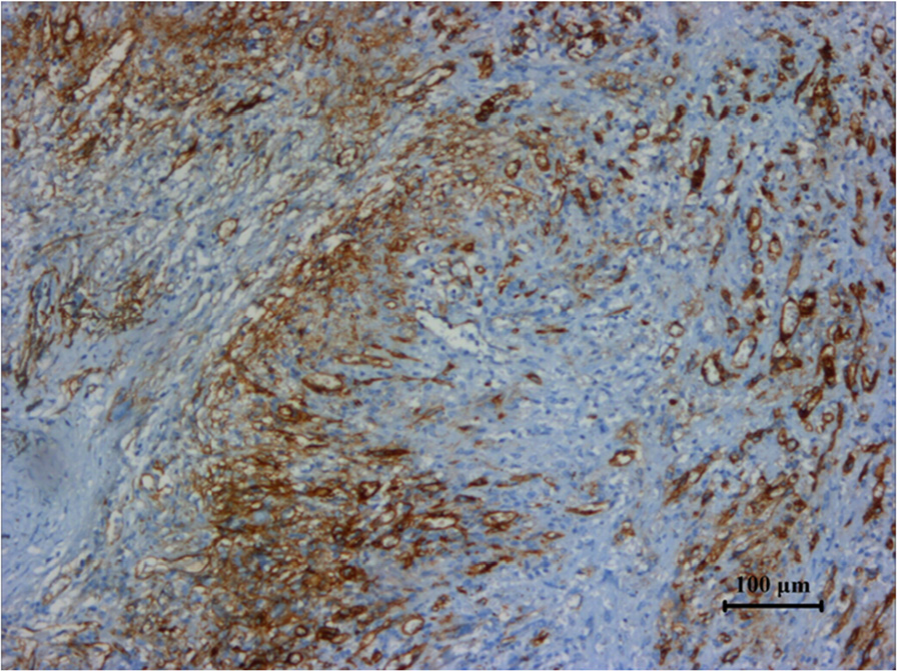
Specimen stained positive for CD34.

**Figure 6:**
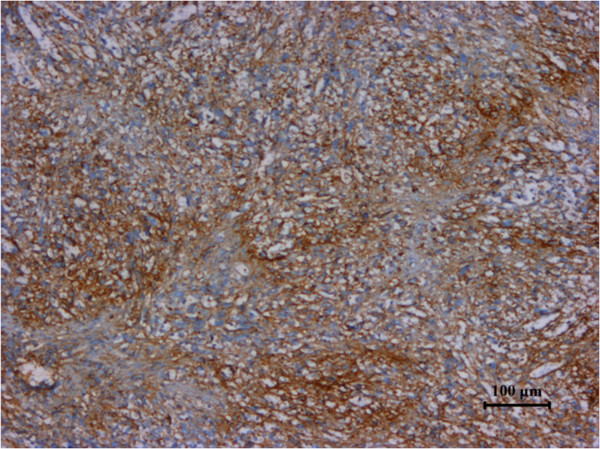
**Specimen stained positive for factor VIII.**

**Figure 7:**
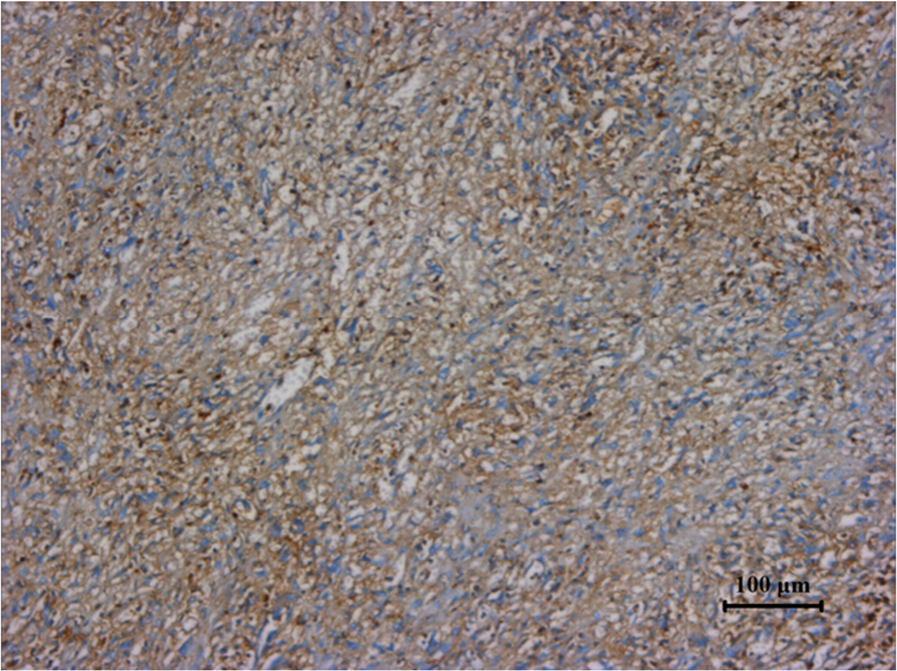
Specimen stained positive for a1ACT.

**Figure 8:**
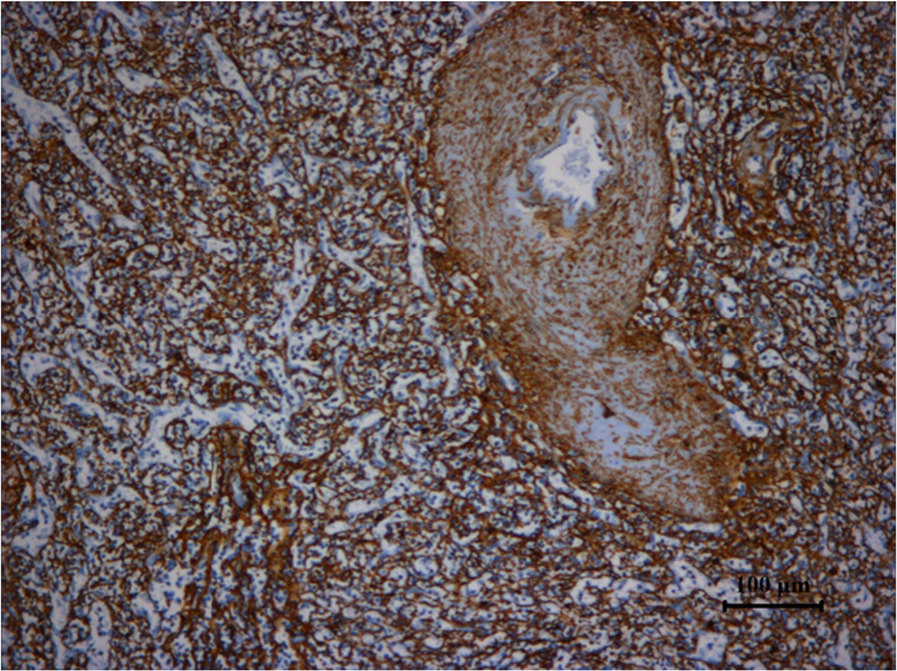
Specimen stained positive for SMA.

**Figure 9:**
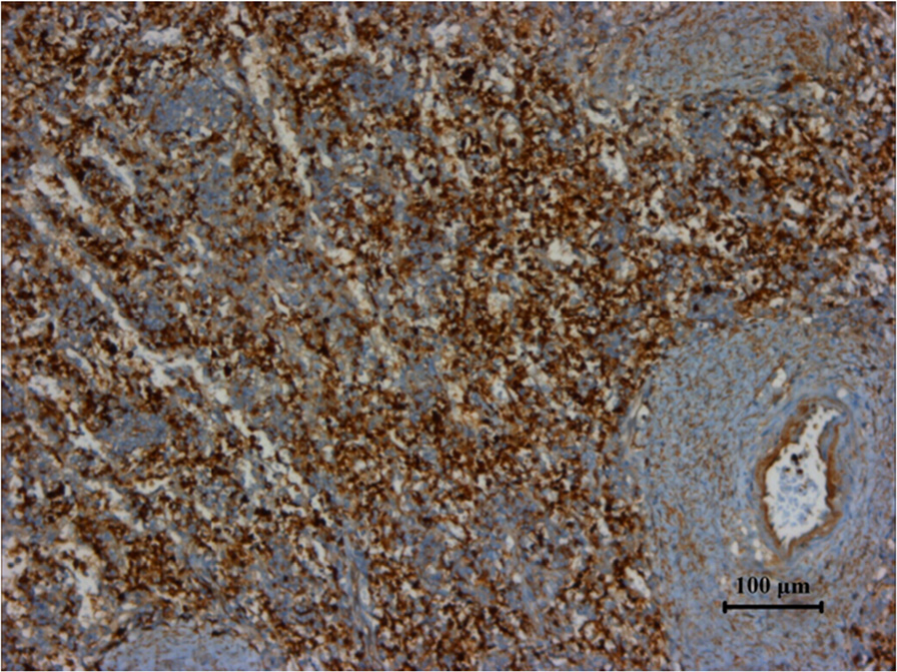
Specimen stained negative for lysozyme.

**Figure 10:**
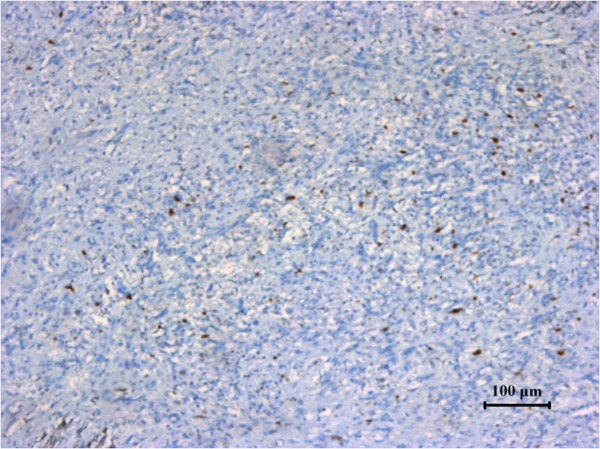
Ki-67 proliferation index less than 10%.

The spleen was 1,650 g in weight, 19 cm × 16 cm × 11 cm in size, with nodular appearance and bleeding. The pathology diagnosis of the excised spleen was angiosarcoma originating from the spleen (Figure 2). Immunohistochemical staining was positive for vimentin (Figure 3), CD31 (Figure 4), CD34 (Figure 5), factor VIII (Figure 6), a1ACT (Figure 7) and SMA (Figure 8), and was negative for lysozyme (Figure 9). The Ki-67 proliferation index was less than 10% (Figure 10).

## Discussion

Angiosarcoma originated from the spleen is an extremely rare malignant tumor, which affects 0.14 to 0.25 per million people [3]. People at any age may develop this disease. The presentation age is between 14 months and 89 years. This neoplasm shows a slight male predominance [3-5]. We reviewed related literatures and summarized the data (Table 1).

**Table 1:** Data of the published literatures regarding splenic rupture secondary to splenic angiosarcoma

**Gender**	**Age**	**Clinical manifestations**	**Laboratory result**	**Early metastasis**	**Treatment**	**Prognosis**	**Reference**
Female	45	Left upper abdominal pain and mass, shock	Anemia, thrombocytopenia	Early multiple metastases	Splenectomy and chemotherapy	Died 4 months after surgery	[6]
Female	79	None	Anemia	No	Splenectomy	Discharged on the 10th postoperative day	[7]
Female	17	Shock, left upper abdominal pain and mass	Anemia, thrombocytopenia	No	Splenectomy	Disease-free of 16 months	[8]
Female	45	Left upper abdominal pain	Thrombocytopenia	Liver metastases after 3 months	Splenectomy and chemotherapy	Died 7 months after surgery	[9]
Male	57	Upper abdominal pain, shock	Anemia, thrombocytopenia	Early liver metastases	Splenectomy	Died 1 day after surgery	[10]
Female	64	Fatigue, upper abdominal pain	Thrombocytopenia	Early bone metastases	Splenectomy and chemotherapy	Died 4 months after surgery	[11]
Male	76	Upper abdominal pain, shock	Anemia, thrombocytopenia	Peritoneal and liver metastases after 4 weeks	Splenectomy	Died 6 weeks after splenectomy	[12]
Female	37	Left upper abdominal pain	Anemia	Liver metastases	Splenectomy and chemotherapy	Died 6 months after surgery	[13]
Male	53	Upper abdominal pain, shock	Anemia, thrombocytopenia	Early liver metastases	Splenectomy	Died 7 months after surgery	[14]
Female	35	Upper abdominal pain, shock	None	Early liver metastases	Splenectomy	Died 4 day after surgery	[15]
Female	80 and over	Shock	Anemia		Splenectomy		[16]
Female	Middle age			Early liver metastases	Splenectomy		[17]

Although the pathogenesis of this tumor is not clear, ionizing radiation, arsenic, vinyl chloride and chemotherapy for lymphoma have been implicated as causative factors [18,19], and it has been reported that the angiosarcoma requires the existence of a benign pathology such as hemangioma or hemangioendothelioma [18,20]. However, there was no evidence of any of these factors involved in our case.

Clinical presentation of splenic angiosarcoma is variable. Left upper abdominal pain is the most common symptom [5]. Other complaints include fatigue, weight loss, and anorexia. Splenomegaly is the most common physical examination finding [5]. Hepatomegaly and left upper quadrant mass are other two common findings. The most serious manifestation is splenic rupture, which often leads to fatal hemorrhage [21].

Laboratory examination can find cytopenia, leukocytosis, thrombocytosis and elevated erythrocyte sedimentation rate. Anemia and thrombocytopenia are presented in more than 50% cases [3,11].

Imaging examinations are helpful for the differential diagnosis, although lacking accuracy. The most common ultrasonography findings are splenomegaly and ill-defined solid and cystic masses in the spleen with heterogeneous echotexture [22,23]. The areas of hemorrhage and necrosis within the tumors are frequently shown as cystic areas. Increased blood flow may be detected in the solid part of the masses on color Doppler images. Computed tomography may reveal an enlarged spleen with hypo-attenuating or hyper-attenuating areas with punctate or massive calcification on plain computed tomography scan (without contrast agent infused) [24,25]. Areas with hyper-attenuation are likely to reflect acute hemorrhage. On contrast-enhanced computed tomography scans, the tumors may exhibit peripheral or heterogeneous enhancement similar to that of hepatic cavernous hemangiomas [26,27]. On magnetic resonance imaging, ill-defined nodular lesions with low or high signal intensity may be seen on both T1-weighted and T2-weighted images. High signal intensity on both T1-weighted and T2-weighted images is related to subacute hemorrhage or tumor necrosis, and low signal intensity is related to chronic hemorrhage or fibrosis within the tumor [28].

Biopsy is contraindicated in splenic angiosarcoma because of high risk of rupture. Histologic studies can therefore only be made after splenectomy. Microscopic examinations reveal freely anastomosing papillary and classical characteristic vascular channels lined by masses of endothelial cells [4]. Immunohistochemically, at least two vascular proliferation markers (CD31, CD34, and factor VII) plus at least one histiocytic differentiation marker (lysozyme and/or CD68) are required to makes the diagnosis [5].

This tumor has a high incidence of early metastasis, as reflected in the reported rates of between 69% and 100%, with the most common sites being in the liver, lungs, bones or bone marrow, lymph nodes, gastrointestinal tract, brain and adrenal glands [3,5,29]. Our patient, however, had metastases in the liver.

Our case died 6 months after the surgery. Primary splenic angiosarcomas are very aggressive neoplasms, with a median survival of 5 months irrespective of treatment. Neuhauser and colleagues reported that 93% of patients died with disseminated tumor within 29 months [5]. Early diagnosis with splenectomy can result in a much more favorable survival rate [3,21], but it is very difficult.

## Conclusions

Splenic angiosarcoma is very rare but has a poor prognosis. It may cause secondary spontaneous rupture, resulting in fatal hemorrhage, which should be paid sufficient attention. Immediate surgery is necessary but the outcome is still unsatisfactory. Attempts should be made to improve the outcome by developing new techniques.

## Consent

Written informed consent was obtained from the patient for publication of this report and any accompanying images.

## Competing interests

The authors declare that they have no competing interests.

## Authors’ contributions

Y-FD and FZ participated in the clinical management of the patient and wrote the manuscript. YJ and C-XW carried out the pathological examination. Y-FD and FZ were involved in the final editing. All authors approved the final manuscript.
